# Single‐Cell RNA‐seq Reveals Deubiquitination Genes as Prognostic Markers in Hepatocellular Carcinoma

**DOI:** 10.1155/ijog/4893924

**Published:** 2026-01-07

**Authors:** Xuening Lv, Chaozhou Chen, Shuxian Zhang, Feng Liang, Qing Zhang

**Affiliations:** ^1^ Department of Gastroenterology, The First People′s Hospital of Lianyungang, Lianyungang, China, lygyy.com.cn; ^2^ Department of Interventional Radiology, Renji Hospital, School of Medicine, Chongqing University, Chongqing, China, cqu.edu.cn; ^3^ Department of Gastroenterology, Huai′an Second People′s Hospital, The Affiliated Huai′an Hospital of Xuzhou Medical University, Huai′an, China, xzmc.edu.cn; ^4^ Department of Hepatology, Huai′an No. 4 People′s Hospital, Huai′an, China

**Keywords:** AI, deubiquitination, HCC, machine learning, prognosis

## Abstract

**Background:**

Hepatocellular carcinoma (HCC) carries a dismal prognosis, yet the contribution of deubiquitination—an essential posttranslational regulator—to its progression remains poorly defined.

**Methods:**

Single‐cell RNA‐seq profiles of 13 treatment‐naïve HCC tumors were integrated with 374 TCGA and 243 ICGC bulk RNA‐seq cohorts. Deubiquitinase (DUB) activity was quantified per cell with AUCell; pathway enrichment was performed with clusterProfiler. A LASSO‐Cox machine learning pipeline was used to build a DUB‐based risk signature, which was cross‐validated internally and externally by time‐dependent ROC analysis.

**Results:**

Malignant cells exhibited divergent DUB transcription relative to immune compartments (myeloid, B). DUB‐high neoplastic subsets displayed heightened inflammatory and IFN‐*γ* signaling, concordant with brisk immune infiltration. A 78‐gene prognostic index robustly stratified survival in discovery and replication cohorts.

**Conclusions:**

This study highlights the role of deubiquitination in HCC progression and its potential as a prognostic biomarker. The developed model could serve as a valuable tool for patient stratification and personalized treatment strategies, although further experimental validation is needed to confirm these findings.

## 1. Introduction

Hepatocellular carcinoma (HCC), the most prevalent form of primary liver cancer, ranks as the second leading cause of cancer‐related mortality worldwide [1]. Currently, treatment options for HCC include surgical resection, liver transplantation, and liver‐targeted therapies [2]. However, only surgical resection and liver transplantation are regarded as potentially curative approaches. Given that the majority of HCC patients are diagnosed at an advanced stage, studies have indicated that merely 15% are eligible for curative treatment [3]. Despite certain advances in targeted therapies, the prognosis for HCC remains dismal due to the emergence of acquired resistance, with a three‐year survival rate of less than 20% and a median overall survival of only 9 months [4]. Moreover, HCC is a highly heterogeneous malignancy, which poses significant challenges for the development of effective therapies and for the accurate prediction of clinical outcomes [5–7]. Consequently, there is a pressing need for further research to systematically investigate the underlying mechanisms of HCC pathogenesis.

Ubiquitination is a prevalent posttranslational modification that orchestrates protein turnover and cellular metabolism [8–11], thereby regulating core processes including the cell cycle, DNA repair, and signal transduction [12–14]. Counterbalancing this pathway, deubiquitination (DUB)—removal of ubiquitin chains from substrate proteins—is indispensable for proteostasis and normal cellular function [15]. Disruption of the equilibrium between ubiquitination and DUB is closely linked to tumorigenesis and progression across cancers, including HCC [16]. Despite extensive investigation of ubiquitination in HCC, the roles, mechanisms, and clinical implications of DUB remain comparatively underexplored [17–19].

With the advancement of high‐throughput sequencing technologies, particularly the advent of single‐cell RNA sequencing (scRNA‐seq), we are now able to investigate the intricate molecular mechanisms of HCC with unprecedented resolution. This study focuses on the role of DUB ‐related genes in HCC, aimed at elucidating their specific contributions to tumor initiation and progression. Through bioinformatic analyses of publicly available datasets, this research seeks to identify potential biomarkers that may enhance prognosis prediction and guide novel therapeutic strategies for HCC patients.

## 2. Methods

### 2.1. Acquisition and Processing of scRNA‐seq Data

This study integrated scRNA‐seq data from 13 HCC samples, which were preprocessed in accordance with the rigorous quality control criteria detailed in the original studies [20]. Data processing was performed using the Seurat R package [21], and cell populations were classified into HCC cells, myeloid cells, NK/T cells, fibroblasts, endothelial cells, B cells, and mast cells. Following data normalization, principal component analysis (PCA) was conducted [22]. To eliminate batch effects, the Harmony R package was employed for correction. Subsequent analyses—including NormalizeData, FindVariableFeatures, ScaleData, RunPCA, FindNeighbors, FindClusters, and RunUMAP—were performed using the Seurat toolkit to achieve precise clustering and molecular characterization of cell subpopulations. Annotation information provided in the original publications was incorporated to support downstream analyses. At the single‐cell level, we quantified DUB activity using AUCell. Cells were stratified into high‐ and low‐score groups based on the median AUCell score [23].

### 2.2. Analysis of Bulk RNA Sequencing Data

Transcriptomic data of HCC patients were obtained from The Cancer Genome Atlas (TCGA; https://portal.gdc.cancer.gov) and an independent cohort from the International Cancer Genome Consortium (ICGC; https://dcc.icgc.org/) was introduced as an external validation dataset to enhance the generalizability and robustness of the model. All transcriptomic data underwent standard normalization procedures, including log‐transformation. To ensure analytical consistency across platforms, the ComBat function from the sva R package was used to adjust for potential batch effects and reduce technical bias [24].

To elucidate key functional pathways and biological mechanisms, gene set variation analysis (GSVA) was conducted for pathway enrichment analysis [25, 26]. High‐quality gene sets were retrieved from the MSigDB database to facilitate comprehensive interpretation of relevant biological processes. The DUB‐related genes were retrieved from the GeneCards database (https://www.genecards.org/) and compiled based on relevance scores.

### 2.3. Construction of a Prognostic Model

Univariate Cox regression analysis was initially performed to systematically identify key genes associated with patient survival. Based on this, a 10‐fold cross‐validation strategy was employed to evaluate the optimal modeling approach from 101 different machine learning algorithm combinations [27]. These included a wide array of survival models such as stepwise Cox regression, Lasso, Ridge, partial least squares Cox regression (plsRcox), CoxBoost, random survival forests (RSF), gradient boosting machines (GBM), elastic net (Enet), supervised principal components (SuperPC), and survival support vector machines (survival‐SVM). The model with the highest concordance index (C‐index), termed the metabolism‐based risk score (MBRS), was ultimately selected as the optimal prognostic tool. The predictive performance of the MBRS was further validated using ROC curve analysis and PCA.

### 2.4. Statistical Analysis

All data processing and statistical analyses were conducted using R software (Version 4.2.0). Kaplan–Meier survival analysis was performed, and statistical significance was assessed using the log‐rank test. For comparisons of continuous variables between groups, either the *t*‐test or Wilcoxon rank‐sum test was applied, depending on data distribution; categorical variables were analyzed using the chi‐square test or Fisher′s exact test. To control for multiple testing errors, false discovery rate (FDR) correction was applied. Pearson correlation analysis was used to evaluate relationships between variables. All statistical tests were two‐sided, and a *p* value < 0.05 was considered statistically significant.

## 3. Results

### 3.1. Sample Integration, Cell Clustering, and Annotation

In this study, 13 human HCC scRNA‐seq samples were retrieved from the GEO database. Initially, Harmony was used to integrate these 13 samples, resulting in a consolidated dataset (Figure 1a). Subsequently, Seurat identified 19 distinct cell clusters (Figure 1b). To enhance the annotation of these clusters, we examined the expression of 16 common marker genes, including EPCAM, PECAM1, VWF, COL1A1, ACTA2, LYZ, CD68, KIT, MS4A2, NKG7, NCR1, MS4A1, CD79A, CD3E, CD8A, and FOXP3. Specifically, the epithelial cell marker EPCAM was predominantly expressed in Clusters 0, 2, 3, 9, and 12; endothelial cell markers PECAM1 and VWF were expressed in Clusters 0 and 10; fibroblast markers COL1A1 and ACTA2 were mainly expressed in Cluster 7; monocyte/macrophage marker LYZ was observed in Clusters 0, 1, 2, 4, 8, 9, 12, 15, and 17; the macrophage marker CD68 was expressed in Clusters 1, 4, 8, 15, and 17; mast cell markers KIT and MS4A2 were predominantly found in Cluster 18; NK and cytotoxic T cell marker NKG7 was primarily expressed in Cluster 6; NCR1, a marker specific to NK cells, was observed in Cluster 6; mature B cell marker MS4A1 was found in Cluster 16; B cell marker CD79A was expressed in Clusters 13 and 16; T cell marker CD3E was identified in Clusters 5, 6, and 11; CD8+ T cell marker CD8A was seen in Clusters 5 and 6; Treg cell marker FOXP3 was expressed in Cluster 11 (Figure 1c).

Figure 1Cell clustering and score of deubiquitinating genes. (a) Integration of 13 samples from hepatocellular carcinoma. (b) Cell clustering by Seurat. (c) The expression of 16 marker genes in cell clusters. (d) Annotation of cell clusters. (e) Scores of deubiquitinating genes in clusters, by AUCell.

**Figure:**
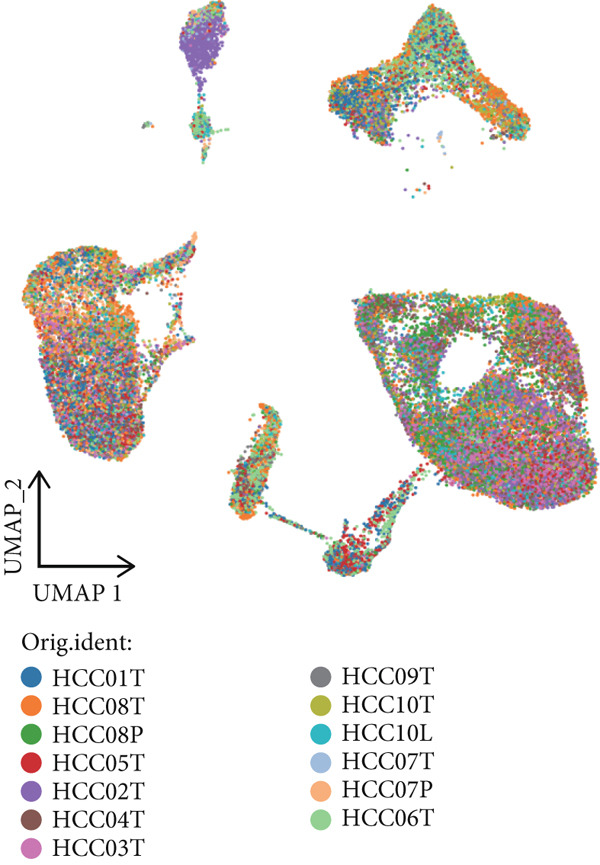
(a)

**Figure:**
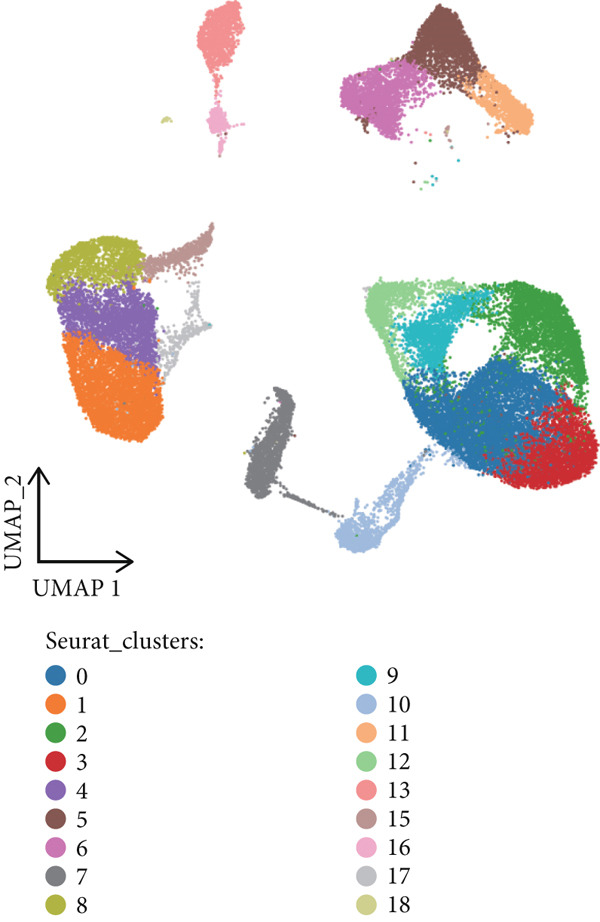
(b)

**Figure:**
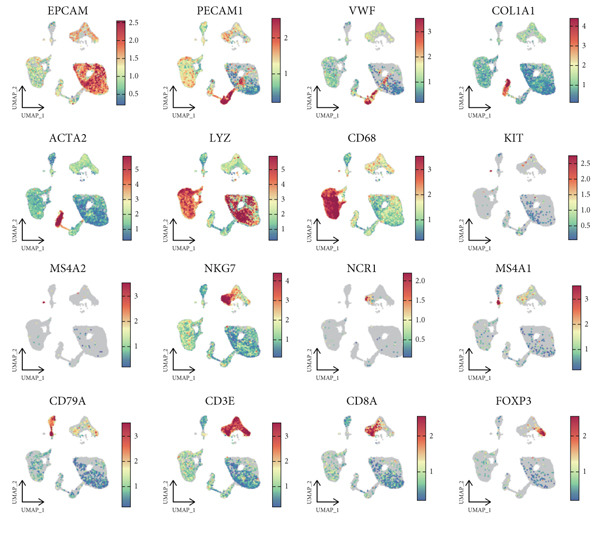
(c)

**Figure:**
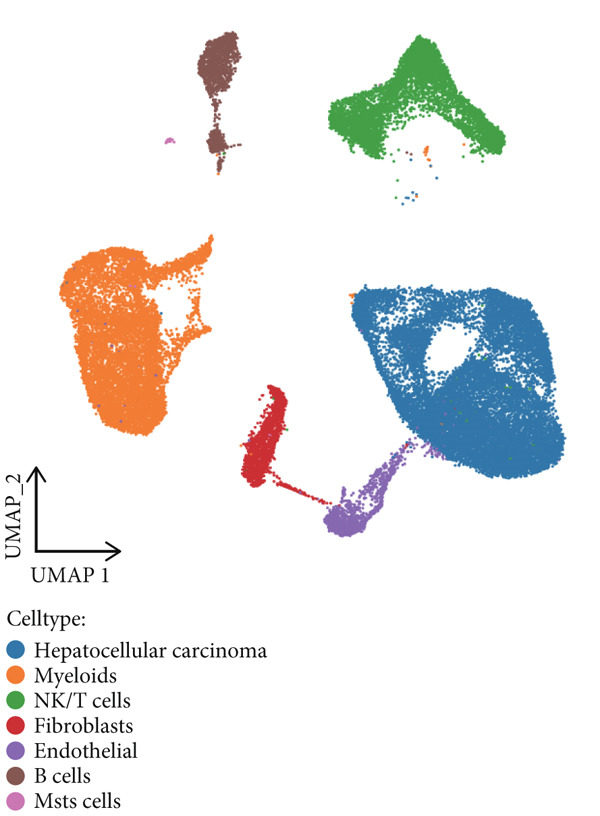
(d)

**Figure:**
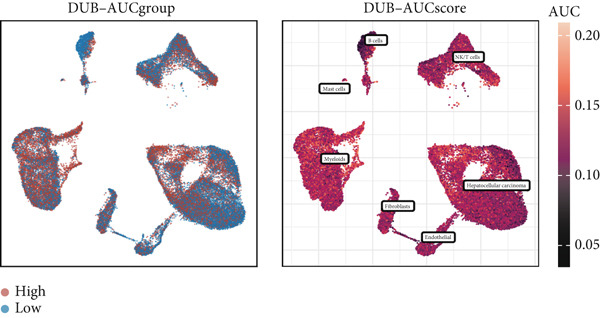
(e)

Finally, based on machine learning annotation results, these cell clusters were classified as HCC, myeloids, NK/T cells, fibroblasts, endothelial, B cells, and mast cells (Figure 1d).

### 3.2. DUB Gene Activity Scoring

A total of 245 DUB genes were selected from the GeneCards database, and activity scores were calculated using AUCell. Cells were divided into high‐score (DUB‐AUC high) and low‐score (DUB‐AUC low) groups based on the median score. DUB‐AUC high cells were primarily found in HCC and myeloids, while DUB‐AUC low cells were predominantly observed in B cells. Other cell types displayed a mixed distribution of high‐ and low‐scoring cells. The results indicated that HCC and myeloids had relatively high scores, suggesting active DUB gene expression, whereas B cells had lower scores compared to other cell types, consistent with the distribution of the high‐ and low‐scoring groups (Figure 1e).

### 3.3. Transcriptomic Differences Between DUB‐AUC High and DUB‐AUC Low Cells

To explore the differences between DUB‐AUC high and DUB‐AUC low cells, analysis was performed using the hallmark gene set from the Broad Institute. These gene sets, well‐curated to encompass key cancer characteristics, are suitable for cancer research. Pathway enrichment analysis revealed that, compared to DUB‐low cells, the majority of tumor‐associated pathways were upregulated in DUB‐high cells, with 23 pathways upregulated, nine with no significant differences, 27 pathways downregulated, and 17 showing no significant differences. The most significantly upregulated pathways included allograft rejection, inflammatory response, interferon gamma response, NF‐*κ*B‐mediated TNF‐*α* signaling, and IL6‐JAK‐STAT3 signaling. Notably, downregulated pathways included the KRAS signaling pathway, skeletal muscle differentiation, bile acid metabolism, and early estrogen response–related signaling pathways (Figure 2a).

Figure 2Similarities and differences between DUB‐high and DUB‐low cells. (a) The difference of GSVA scores. (b) The correlation of cell clusters in DUB‐low cells. (c) The correlation of cell clusters in DUB‐high cells. (d) Ro/e (representation over expected) analysis comparing cell type enrichment between tissues with high and low DUB‐pathway activity. Bar length indicates the degree of enrichment; cell types with Ro/e > 1 are considered enriched in the corresponding group.

**Figure:**
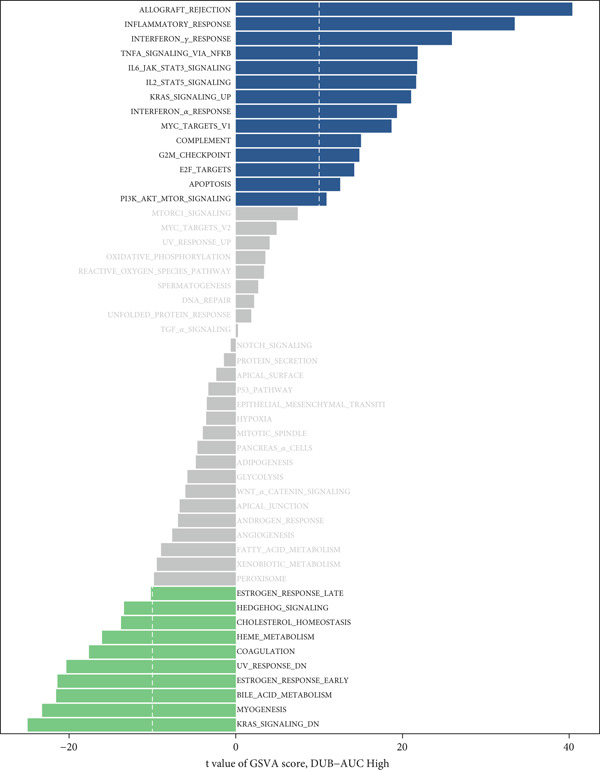
(a)

**Figure:**
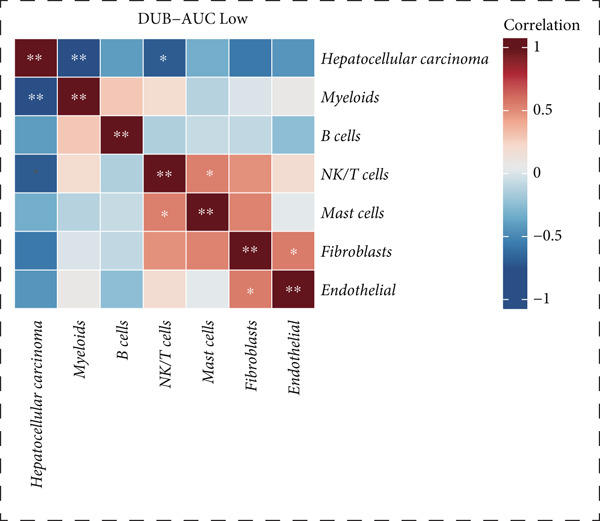
(b)

**Figure:**
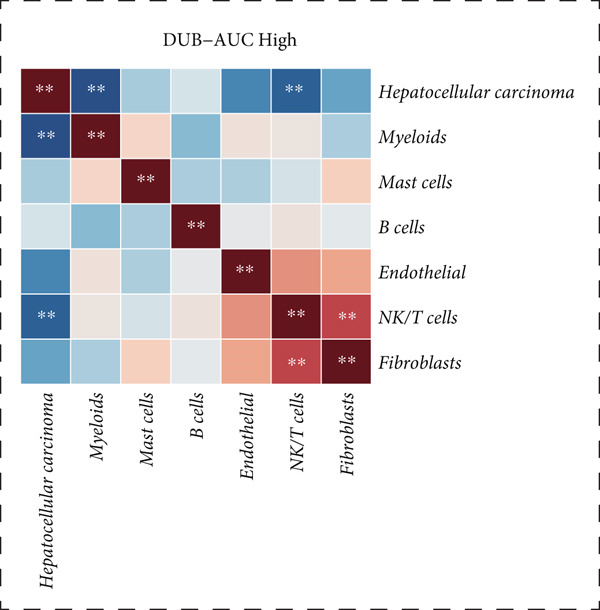
(c)

**Figure:**
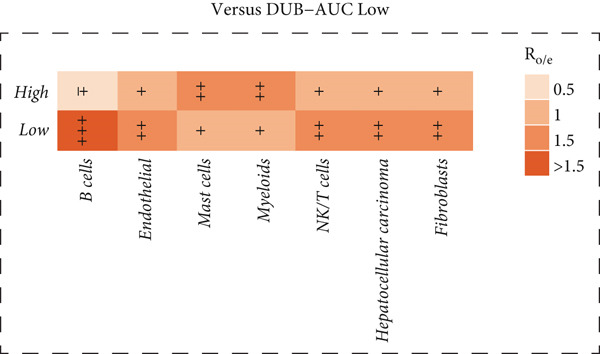
(d)

Correlation and clustering analysis revealed that, in DUB‐AUC low cells, mast cells were significantly correlated with NK/T cells (*p* < 0.05), and endothelial cells were correlated with fibroblasts (*p* < 0.05). Interestingly, HCC showed the weakest correlation with myeloids and NK/T cells, especially with myeloids. No significant correlations were found in other combinations. In DUB‐AUC high cells, NK/T cells were strongly correlated with fibroblasts (*p* < 0.01) (Figure 2b,c).

Ro/e (representation over expected) analysis showed that B cells were significantly enriched in the DUB‐AUC low group, while they were notably reduced in the DUB‐AUC high group (Figure 2d). Overall, this revealed significant differences in the activity of various cell types between the two groups, providing a basis for studying changes in cell type activity.

### 3.4. Differences in Intercellular Communication Between the Two Groups

To examine intercellular communication differences between DUB‐AUC high and DUB‐AUC low cells, communication analysis was performed. The results indicated that the number of cell communications in the DUB‐AUC high group was significantly lower than that in the DUB‐AUC low group. However, the communication strength was higher in the DUB‐AUC high group than in the DUB‐AUC low group (Figure 3a). Seven subtypes of cells in the DUB‐low group exhibited more active communication behavior, with higher frequencies of bidirectional communication than those in the DUB‐high group (Figure 3b). To further identify significantly differential signaling pathways, the information flow between the two groups was compared, revealing that SPP1, CD99, and MK pathways were significantly downregulated in DUB‐low cells (Figure 3c).

Figure 3Cell–cell communication analysis between DUB‐high and DUB‐low cells. (a) Number of inferred interactions and interaction strength in DUB‐high and DUB‐low cells. (b) Interaction strength of eight cell clusters in DUB‐high and DUB‐low cells. (c) Information flow in DUB‐high and DUB‐low cells.

**Figure:**
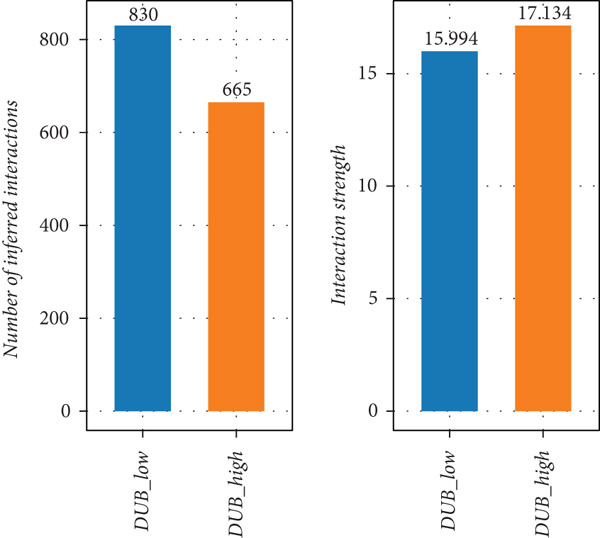
(a)

**Figure:**
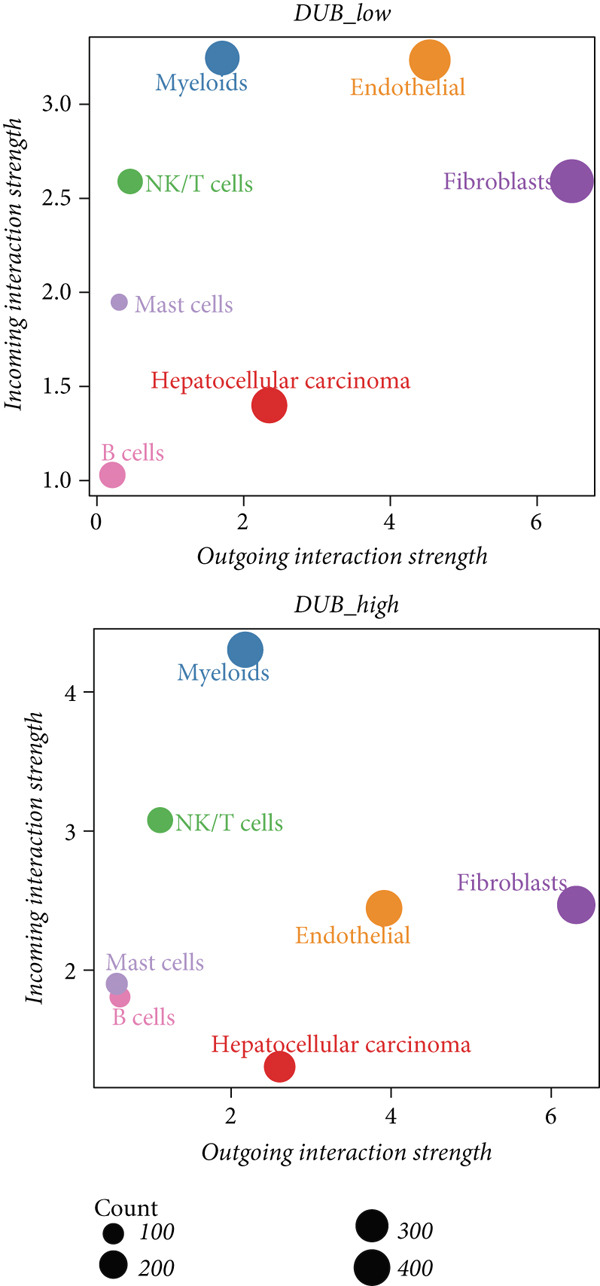
(b)

**Figure:**
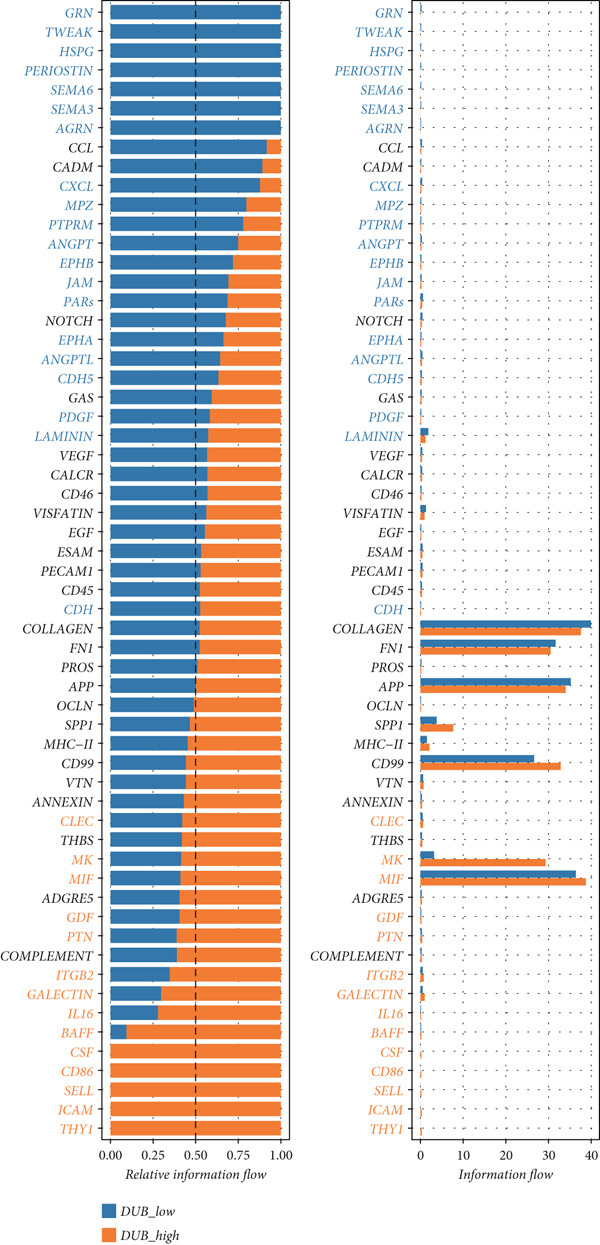
(c)

### 3.5. Identification of DUB‐Associated Genes

To investigate the gene expression characteristics associated with DUBs and clinical prognostic factors, multiple bioinformatics analyses were conducted. Firstly, PCA was performed to integrate samples from the ICGC and TCGA datasets, and no significant batch effects were observed (Figure 4a). (Figure 4b) shows the spatial distribution of different genes across various chromosomes. Subsequently, univariate Cox regression analysis was performed on both cohorts to identify genes with prognostic significance (*p* < 0.01). These genes were further filtered to satisfy differential expression conditions in HCC tissues (|log2FC| > 0.5, adjusted *p* < 0.05), resulting in the identification of 78 significantly prognostic genes, 52 of which were associated with poor prognosis (e.g., YBX1 (Y‐box binding protein 1), RAN, and CAP1) and 26 with favorable prognosis (e.g., CYP2C9, PON1, and CFHR1) (Figure 4c). Additionally, Kyoto Encyclopedia of Genes and Genomes (KEGG) enrichment analysis revealed that these genes were involved in several biological processes, cellular components, and molecular function pathways, such as Fc gamma R–mediated phagocytosis and bacterial invasion. Gene Ontology (GO) analysis further revealed their enrichment in various biological processes (e.g., secretory granule lumen), cellular components (e.g., collagenous extracellular matrix), and molecular functions (e.g., endopeptidase activity), suggesting their crucial role in these functional categories (Figure 4d).

Figure 4Development of a prognostic signature. (a) Sample distribution across datasets. (b) Circos plot illustrating genomic alterations and the distribution of significant genes across chromosomes in the TCGA and ICGC datasets. (c) Univariate COX regression analysis of DUB genes. (d) Kyoto Encyclopedia of Genes and Genomes (KEGG) and Gene Ontology (GO) enrichment analyses performed on DUB‐associated genes.  ^∗^ *p* < 0.05,  ^∗∗^ *p* < 0.01, and  ^∗∗∗^ *p* < 0.001.

**Figure:**
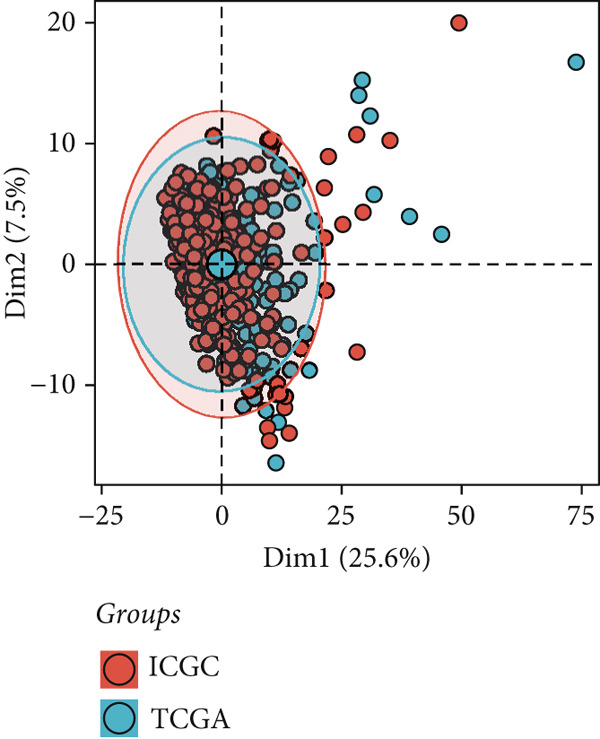
(a)

**Figure:**
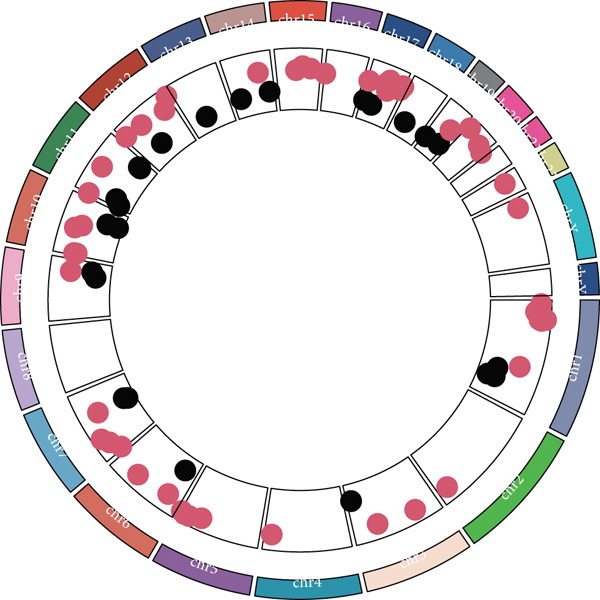
(b)

**Figure:**
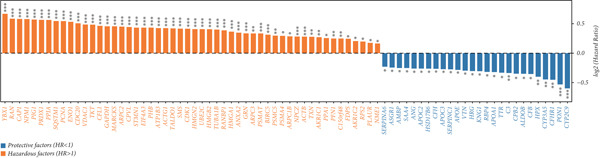
(c)

**Figure:**
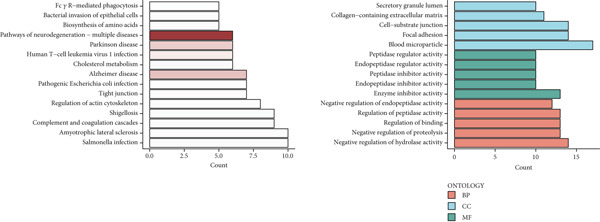
(d)

### 3.6. Construction of Prognostic Models and Relationship With Immune Microenvironment

To develop a consistent DUB‐associated signature (DAS) model, 101 machine learning algorithms were employed to evaluate the 78 genes. Using TCGA as the training set and ICGC as the validation set, 10‐fold cross‐validation was applied to construct 101 predictive models, and the C‐index was calculated (Figure 5a). The StepCox[both] + RSF model, which exhibited the highest average C‐index, was selected and demonstrated strong performance in both the training and validation sets. Survival analysis was conducted using median risk scores as the cutoff, revealing significantly poorer prognosis for high‐risk patients in both datasets (Figure 5b). ROC curve analysis was performed to evaluate model prediction capability (Figure 5c): in TCGA‐HCC, the 1‐year, 3‐year, and 5‐year AUCs were 0.95, 0.94, and 0.95, respectively; in the ICGC validation set, the AUCs for 1, 3, and 5 years were 0.74, 0.77, and 0.43, respectively. The model exhibited strong predictive power at 1 and 3 years, with TCGA‐HCC showing superior predictive ability over ICGC. The gene expression patterns shown in Figure 5d effectively distinguished samples with different risk scores, further validating the importance of these genes in varying risk groups. Finally, the correlation between the expression of DUB‐related genes and risk scores (riskScore) was evaluated, revealing significant positive correlations for genes such as YBX1, PRDX1, SQSTM1, PTTG1, STMN1, and FDPS, and a negative correlation with PON1 (Figure 6a). As risk scores increased, the expression levels of these genes generally rose. To explore immune microenvironment differences, the expression of MHC I, MHC II, other MHC, costimulatory molecules, and coinhibitory molecules was further analyzed. The results indicated that these immune marker genes were expressed at higher levels in DUB‐low samples, with particularly significant differences observed in costimulatory and coinhibitory molecule‐related genes (Figure 6b).

Figure 5Construction and validation of the signature. (a) A consensus DUB‐Associated signature, by 101 machine‐learning algorithms. (b) Survival curve of high‐risk samples and low‐risk samples across two datasets. (c) Area under curve (AUC) values of the DUB‐associated signature in predicting 1‐, 3‐, and 5‐year survival rates. (d) PCA plots for the low‐risk and high‐risk groups based on the risk score.

**Figure:**
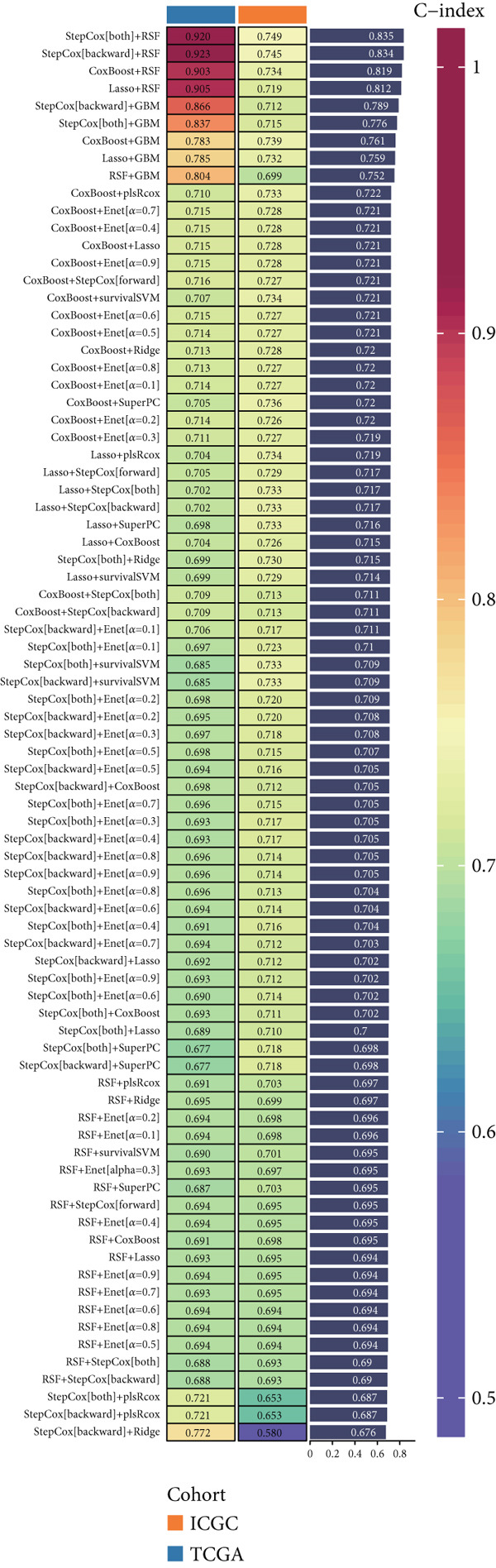
(a)

**Figure:**
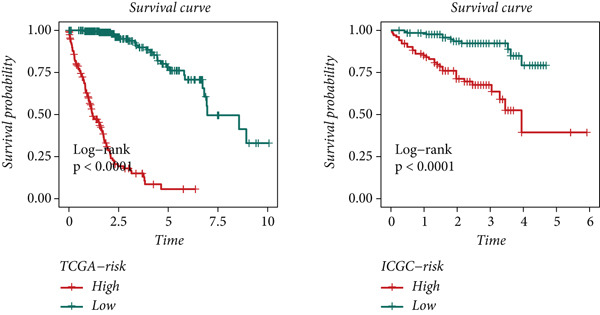
(b)

**Figure:**
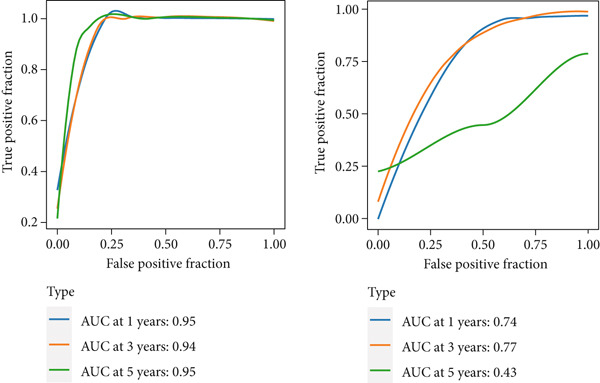
(c)

**Figure:**
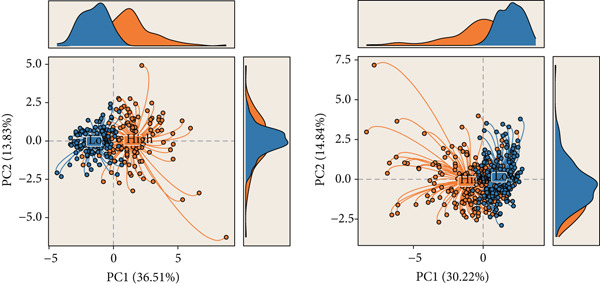
(d)

Figure 6Correlation analysis and risk score evaluation for survival prediction. (a) Correlation analysis between risk scores and DUB‐Associated genes. (b) The expression of MHC, coinhibitors, and costimulators in high‐risk samples and low‐risk samples.

**Figure:**
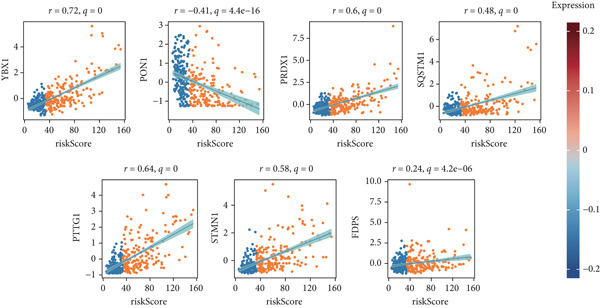
(a)

**Figure:**
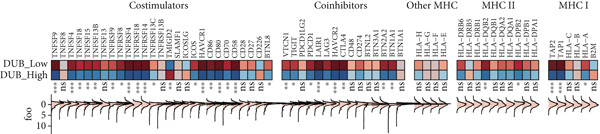
(b)

## 4. Discussion

HCC ranks among the Top 6 most prevalent cancers worldwide [28]. This study reveals significant variations in the activity driven by DUB genes within HCC. Tumor cells with high expression of DUB‐associated genes exhibit enhanced tumor‐promoting pathways, and the high expression of DUB genes is predominantly enriched in myeloid cells and mast cells. In contrast, cells with low DUB expression are more enriched in B cells and NK cell phenotypes. Thus, the prognostic risk feature model constructed based on DUB expression levels holds substantial clinical predictive value.

Additionally, this study found that in DUB‐high expression cells, pathways such as the inflammatory response, interferon *γ* response, NF‐*κ*B‐mediated TNF‐*α* signaling, and IL6‐JAK‐STAT3 signaling were upregulated. These pathways are closely linked to various immune cells, including CD8 T cells, CD4 T cells, macrophages, neutrophils, and NK cells. This suggests a higher degree of immune cell infiltration in DUB‐high expression cells and implies that DUB genes may be involved in immune killing and immune escape mechanisms in hepatocytes, positioning them as potential therapeutic targets.

Cellular communication analysis indicated that DUB‐high expression cells exhibit stronger interaction intensities, suggesting that DUB genes may play a role in enhancing intercellular communication. Further information flow analysis revealed that in DUB‐high expression cells, SPP1, MK (Midkine), and CD99 were highly expressed. SPP1 is a secreted protein closely related to macrophages [29]; CD99 is a cell membrane protein predominantly found in various immune cells [30]; Midkine, a neurotrophic factor, is crucial during embryonic development and plays a role in cell migration, proliferation, survival, repair, and angiogenesis [31]. These findings highlight the more active cellular communication and immune cell activity in DUB‐high expression cells, warranting further investigation.

Through univariate Cox analysis, this study identified 78 DUB‐related genes significantly associated with prognosis. These genes are closely linked to the cell cycle, transcriptional regulation, tumor cell migration, invasion, metabolism, antioxidant activity, and immune regulation. Among them, YBX1 is a highly conserved multifunctional protein found in both the nucleus and cytoplasm, with DNA/RNA‐binding capacity. Previous research has indicated that YBX1 can promote homologous recombination by recognizing m5C modifications, leading to platinum drug resistance in ovarian cancer [32]. PON1 is a serum esterase with antioxidant and anti‐inflammatory functions, which induces the metastasis of lung cancer cells through its antioxidant activity [33]. In this study, PON1 was associated with favorable prognosis, highlighting its heterogeneous role across different cancers. PRDX1 (Peroxiredoxin 1) is a widely expressed peroxidase, and in prostate cancer, HJURP may inhibit tumor cell sensitivity to iron mutation inducers via the PRDX1 pathway [34]. SQSTM1 (Sequestosome 1), also known as p62, is a multifunctional adaptor protein involved in processes such as autophagy, oxidative stress responses, protein degradation, inflammation, and tumor progression [35, 36]. PTTG1 (pituitary tumor‐transforming gene 1) encodes a small molecule protein, securin, which promotes pancreatic cancer cell proliferation by regulating c‐myc [37]. STMN1 (Stathmin 1) is a microtubule‐binding protein associated with breast tumor progression and paclitaxel resistance [38]. FDPS (Farnesyl Diphosphate Synthase) is a key rate‐limiting enzyme in the mevalonate pathway. Previous studies have shown that FDPS is significantly overexpressed in glioma tissues and is associated with poor prognosis, promoting tumor growth [39]. These findings emphasize the need for further exploration of these genes′ roles in immune regulation in HCC.

The DAS model demonstrated excellent predictive power for the prognosis of HCC patients, establishing itself as an effective tool for assessing patient survival risk. This model enables precise patient stratification based on predicted outcomes, providing a foundation for personalized treatment approaches and guiding the development of more rational treatment strategies. Additionally, it can assist in identifying patients at high risk of disease progression, prompting more proactive clinical interventions based on their risk status. The clinical application of this model is expected to enhance decision‐making efficiency, optimize treatment management, and ultimately improve patient prognosis and quality of life.

This study integrates scRNA‐seq and bulk RNA sequencing data to systematically analyze the expression profiles of DUB ‐related genes and their roles in intercellular signaling and immune regulation. Despite some limitations, such as the primary reliance on publicly available data and the lack of clinical sample validation, which may restrict the generalizability of the results to specific populations, and the fact that some inferences remain at the computational analysis level without experimental validation, the innovative nature of this study is noteworthy. It provides valuable references for subsequent experimental research and mechanistic exploration.

## 5. Conclusion

The study explores the role of DUB‐related genes in HCC and their potential as prognostic biomarkers. We found that high DUB gene expression (DUB‐high) is associated with upregulated immune‐related pathways and increased immune cell infiltration. A prognostic model based on DUB expression levels was developed, showing strong predictive power for patient survival. This model offers a valuable tool for risk stratification and personalized treatment in HCC. Despite some limitations, such as the need for experimental validation, the findings highlight DUB′s crucial role in HCC progression and its potential as a therapeutic target. Further research is needed to validate these results and refine the prognostic model.

## Disclosure

All the authors read and approved the final manuscript.

## Conflicts of Interest

The authors declare no conflicts of interest.

## Author Contributions

X.L. and C.C. drafted the initial manuscript. X.L. and C.C. performed the literature search and collected the data. X.L. and C.C. were responsible for data analysis. Q.Z., F.L., and S.Z. supervised the project and designed the experiments. X.L. and C.C. have contributed equally to this work.

## Funding

This work was supported by the Digestive Tract Early Cancer Physician Co‐growth Program of the National Cancer Screening Center, GTCZ‐2022‐JS‐32‐0002; General Project of Lianyungang Municipal Health Commission, 202309; 2023 Huai’an Basic Research Program (Joint Special Project), HABL2023074; and 2024 Huai’an Science and Technology Plan Project, HAB202425.

## Data Availability

This study used public data from an online database. The data can be obtained freely from TCGA (https://www.cancer.gov/ccg/research/genome-sequencing/tcga) and GEO (https://www.ncbi.nlm.nih.gov/geo/).
